# Tumor Necrosis Factor-α-Induced Ototoxicity in Mouse Cochlear Organotypic Culture

**DOI:** 10.1371/journal.pone.0127703

**Published:** 2015-05-22

**Authors:** Qian Wu, Guo-Peng Wang, Jing Xie, Jing-Ying Guo, Shu-Sheng Gong

**Affiliations:** 1 Department of Otolaryngology, Head and Neck Surgery, Beijing Tongren Hospital, Capital Medical University, Beijing, 100730, China; 2 Department of Otolaryngology, Head and Neck Surgery, Beijing Friendship Hospital, Captital Medical University, Beijing, 100050, China; Harvard University, UNITED STATES

## Abstract

Tumor necrosis factor (TNF)-α is a cytokine involved in acute inflammatory phase reactions, and is the primary upstream mediator in the cochlear inflammatory response. Treatment of the organ of Corti with TNF-α can induce hair cell damage. However, the resulting morphological changes have not been systematically examined. In the present study, cochlear organotypic cultures from neonatal mice were treated with various concentrations and durations of TNF-α to induce inflammatory responses. Confocal microscopy was used to evaluate the condition of hair cells and supporting cells following immunohistochemical staining. In addition, the ultrastructure of the stereocilia bundle, hair cells, and supporting cells were examined by scanning and transmission electron microscopy. TNF-α treatment resulted in a fusion and loss of stereocilia bundles in hair cells, swelling of mitochondria, and vacuolation and degranulation of the endoplasmic reticulum. Disruption of tight junctions between hair cells and supporting cells was also observed at high concentrations. Hair cell loss was preceded by apoptosis of Deiters’ and pillar cells. Taken together, these findings detail the morphological changes in the organ of Corti after TNF-α treatment, and provide an *in vitro* model of inflammatory-induced ototoxicity.

## Introduction

Inflammation plays a critical role in the pathogenesis of many types of sensorineural hearing loss and tinnitus. Inflammatory responses occur in the inner ear in a variety of pathologic conditions, such as noise-induced hearing loss [1, 2], and inflammation is associated with hearing loss following bacterial meningitis [3], autoimmune neurosensory hearing loss, idiopathic sudden sensorineural hearing loss [4], and cisplatin ototoxicity [5]. Within the cochlea, various cell types, including spiral ligament type I fibrocytes, outer hair cells (OHCs), and supporting cells, can release tumor necrosis factor (TNF)-α, a pro-inflammatory cytokine capable of initiating apoptosis [1, 6, 7].

TNF-α is considered the prominent primary upstream mediator of the inflammatory response [8], for which TNF receptor 1 is the major signaling receptor. Binding of TNF-α induces receptor trimerization and recruitment of TNF receptor 1-associated death domain protein, which serves as a platform to trigger complex signaling cascades and networks. One such cascade involves caspases, such as caspase-3, which are responsible for the proteolytic cleavage of a broad spectrum of cellular targets, ultimately leading to apoptosis [9].

Intracochlear perfusion of TNF-α increases hearing threshold, whereas blockade of TNF-α significantly reduces post-meningitic hearing loss and cochlear injury [3]. Perfusion of TNF-α in the scala tympani of guinea pigs leads to leukocyte infiltration around the venules and within scala tympani [10]. *In vitro*, excessive levels of TNF-α increase the expression of apoptosis-related genes and induce cochlear cell death [11, 12]. The apoptotic process occurs as a result of mitochondrial fission and nitric oxide production in the auditory cell line [13].

Incochlear organotypic cultures, TNF-α ototoxicity to hair cells is dose-dependent [12]. However, there have been no systematic studies to examine the associated morphologic changes. Thus, the aim of the present study was to examine the morphologic and ultrastructural changes in the organ of Corti in response to TNF-α treatment using an organotypic culture model from neonatal mice.

## Materials and Methods

### Cochlear organotypic cultures and TNF-α-induced dose- and time-dependent curves

The care and use of animals were in accordance with the published code of practice for the use of animals in research by the Chinese National Health and Medical Research Council and were approved by the Beijing Ethics Committee for Animal Usage (China).

Postnatal-day 3 CD-1 mouse pups were euthanized by decapitation and the cochleae were dissected out. The basilar membrane together with the organ of Corti and spiral ganglion neurons was isolated as described elsewhere [14]. A drop (20 μL) of cool, rat tail collagen type I gel (Cat. No. 40236; Collaborative Biomedical Products, Bedford, MA, USA) in 10 × Basal Medium Eagle (Cat. No. B9638; Sigma-Aldrich, St. Louis, MO, USA) with 2% sodium carbonate (Cat. No. S2127; Sigma-Aldrich) (9:1:1 ratio) was placed in a 35-mm culture dish (Cat. No. 430165; Corning Inc., Corning, NY, USA) and allowed to gel at room temperature. Then, 1.3 mL of serum-free medium was added, consisting of: 2 g bovine serum albumin (Cat. No. BAH-1108; Equitech-BIO, Kerrville, TX, USA), 2 mL 100 × Insulin-Transferrin-Selenium-X supplement (Cat. No. 51500–056; Invitrogen of Thermo Fisher Scientific, Waltham, MA, USA), 4.8 mL of 20% glucose (Cat. No. G7021; Sigma-Aldrich), 0.4 mL penicillin G (8.8 U/mL) (Cat. No. 0242-100MU; Amresco LLC, Solon, OH, USA), and 2 mL of 200 mM glutamine (Cat. No. G-8540; Sigma-Aldrich) in 190.8 mL of 1 × Basal Medium Eagle (Cat. No. B1522; Sigma-Aldrich) [15]. The basilar membrane was placed on the surface of the collagen gel. The culture was placed in an incubator (Thermo Fisher Scientific) and maintained at 37°C in 5% CO_2_. The culture medium was replaced with 2 mL fresh culture medium with or without various concentrations of TNF-α the following day, and replaced every 2 d.

For the dose-time-dependent studies, the cochleae were randomly divided into 12 groups (*n* ≥ 6 per condition). A stock solution of TNF-α (Cat. No. PMC3013; Invitrogen), which dilutions were made in sterile, distilled water, was added to the culture medium for final concentrations of 0.5, 5.0, and 20.0 μg/mL. In the control group, we added the same volume of sterile, distilled water to the SFM. Control and TNF-α-treated groups were harvested for analysis after 2, 4, or 8 d.

### Immunofluorescence staining and confocal microscopy

The cochlear explants were fixed using 4% formalin (Cat. No. SF100-20; Thermo Fisher Scientific) in phosphate-buffered saline (PBS) at 4°C overnight. After washing with PBS, the explants were treated with 0.3% Triton X-100 in PBS for 30 min, and then blocked using 0.1% bovine serum albumin and 10% normal goat or donkey serum for 1 h at room temperature. We used primary antibodies against myosin 7a (diluted 1:200) (Cat. No. 138–1; Developmental Studies Hybridoma Bank at the University of Iowa, Iowa City, IA, USA) and cleaved caspase-3 (diluted 1:200) (Cat. No. 9661; Cell Signaling Technology, Danvers, MA, USA). Alexa Fluor 488-conjugated phalloidin (diluted 1:300) (Cat. No. A12379; Invitrogen) was used to label F-actin. After rinsing in PBS three times, Alexa Fluor 488-, 568- or 647-conjugated (diluted 1:300) (Invitrogen) secondary antibodies were used along with DAPI (diluted 1:500) (Cat. No. D1306; Invitrogen) for nuclear visualization. The specimens were mounted on glass slides with Fluromount-G (Cat. No. 0100–01; SouthernBiotech, Birmingham, AL, USA). Images were acquired using epifluorescence (Tis-U; Nikon Corp., Tokyo, Japan) with a 60 × objective lens or by confocal microscopy (TCS SP5 II; Leica Microsystems, Wetzlar, Germany) with a 63 × objective and analyzed with Photoshop CS4 (Adobe Systems, San Jose, CA, USA).

### Scanning electron microscopy (SEM)

Organotypic cultures were fixed for 15 min with 2.5% glutaraldehyde in 0.1 M sodium cacodylate buffer, pH 7.4, containing 2 mM CaCl_2_, washed in phosphate buffer (PB), and then post fixed for 10 min with 1% OsO_4_ in the same buffer and washed. The tissues were dehydrated through an ethanol series, critical-point dried by CO_2_ (EM CPD300; Leica) and sputter-coated with platinum. The samples were examined on a field-emission scanning electron microscope (S-4800; Hitachi, Ltd., Tokyo, Japan).

### Transmission electron microscopy (TEM)

The explants were fixed for 2 h with 2.5% glutaraldehyde in 0.1 M PB (pH 7.4) at room temperature, then washed in phosphate buffer. After post fixation for 1 h with 1% OsO_4_ in the same buffer, samples were dehydrated through a graded ethanol series and embedded in Spurr’s epoxy resin via propylene oxide. Ultra-thin sections were stained with uranyl acetate and lead citrate and mounted on grids. Grids were examined using a transmission electron microscope (JEM-2100; JEOL, Tokyo, Japan).

### Cochleogram and hair cell counts

The number of hair cells was counted in 166.7-μm intervals along the entire length of the cochlea from base to apex under an epifluorescence microscope, and the loss of hair cells was calculated as a percent for each interval. A cochleogram was constructed showing the percentage hair cell loss as a function of the distance from the apex of the cochlea [16].

Alexa Fluor 488-conjugated phalloidin was used to label stereocilia bundles and cuticular plates. Hair cell loss was counted if both the stereocilia and the cuticular plate were absent. All quantitative analyses were independently performed in a blind fashion by two investigators, and both sets of data were averaged.

### Statistical analysis

Data were analyzed and converted into cochleogram using GraphPad Prism 5 (GraphPad Software Inc., La Jolla, CA, USA). The data were shown as the mean ± standard deviation. Differences were evaluated using the Student’s *t*-test, and *p <* 0.05 was considered to be statistically significant.

## Results

### TNF-α-induced stereocilia bundle damage

**Fig 1:**
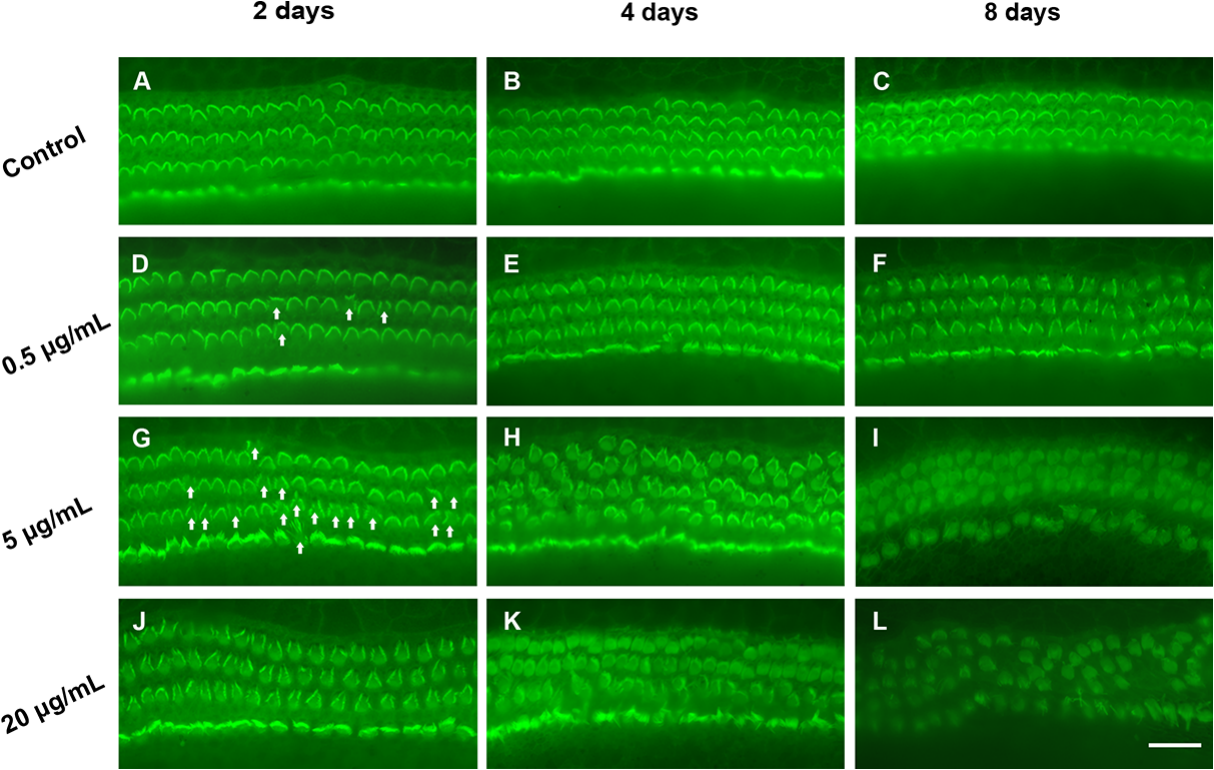
Immunofluorescence images of stereocilia bundles in cultured organs of Corti. A–C) In control cultures, V-shaped stereocilia bundles are arranged orderly on outer hair cells. D–L) Treatment with tumor necrosis factor-α disrupts stereocilia bundles, with damage to the top of V-shaped bundles (arrows) observed. Stereocilia fusion and loss, and elimination of the cuticular plate were observed with the increase in dose and duration. Scale bar: 25 μm.

The effects of various concentrations of TNF-α on organ of Corti viability were examined at different time points. TNF-α induced ototoxicity to the stereocilia bundle was assessed by phalloidin labeling within a series of confocal images from the middle turn of the cochlea (approximately 60% of the distance from the apex) with the focal plane along the height of the stereocilia bundle. Three rows of OHCs and one row of inner hair cells (IHCs) were visible in control groups cultured for 2, 4, or 8 d (Fig 1A–1C). The alignment of OHCs indicated by their V-shaped bundles indicates that the organization of the organ of Corti was well maintained at this stage and the stereocilia bundle integrity was maintained. In contrast, minor damage to stereocilia was observed in the cultures treated with 0.5 μg/mL TNF-α for 2 d (Fig 1D), which was more prominent at a higher dose (5 μg/mL), particularly at the top of their V-shaped bundles (Fig 1G, arrows).

Hair cells presented an increasingly disorderly arrangement after 4 d with 0.5, 5.0, and 20.0 μg/mL TNF-α (Fig 1E, 1H and 1K, respectively). Fusion and absence of bundles was observed after 8 d with TNF-α treatment, along with an elongation of some (Fig 1I). Some OHC stereocilia and the cuticular plate disappeared and IHC stereocilia fused into giant stereocilia after 8 d with 20 μg/mL TNF-α (Fig 1L).

**Fig 2:**
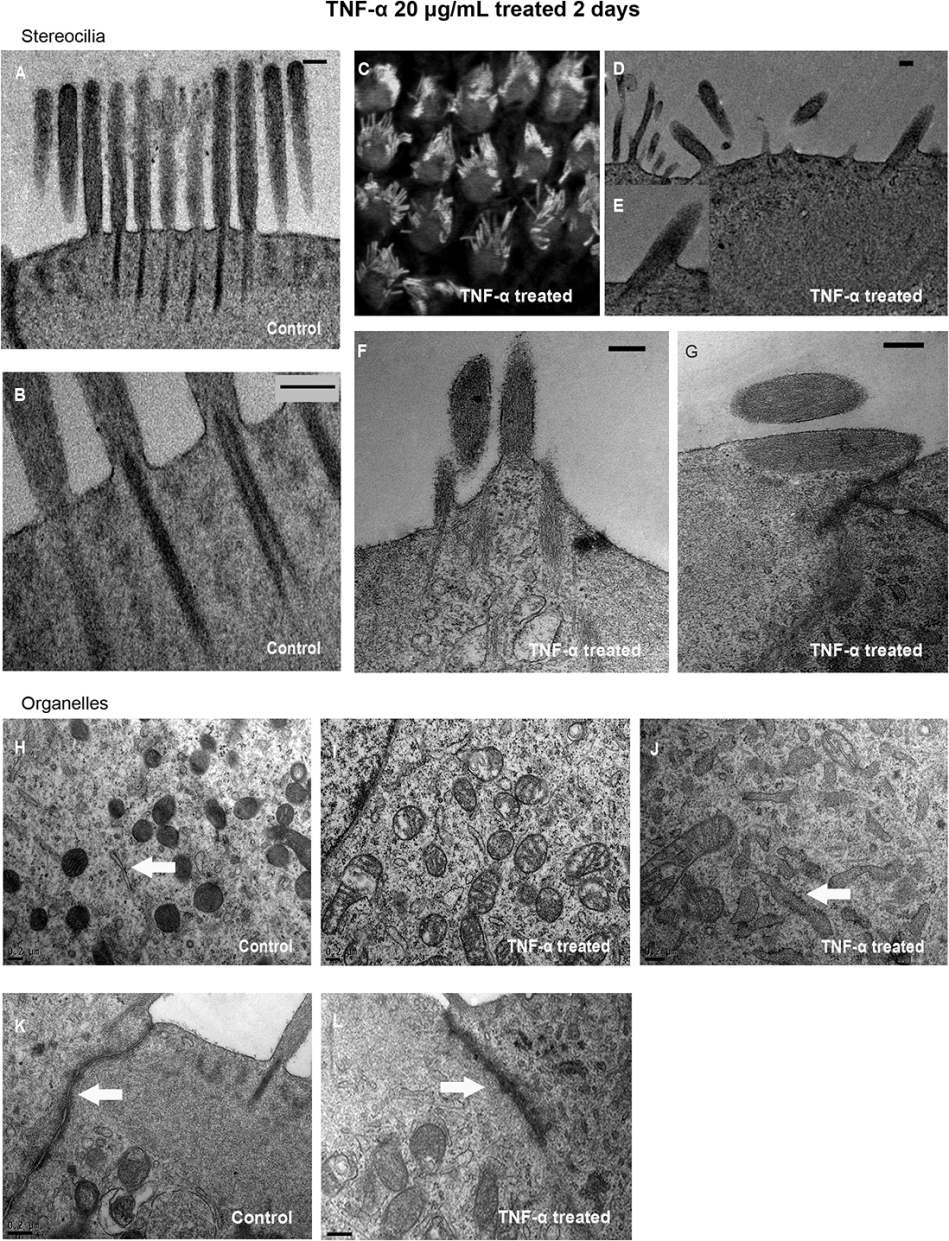
Transmission electron microscopy of stereocilia bundles and organelles of organ of Corti. A,B) Bundles of stereocilia in outer hair cells are arranged orderly and central filaments are straightly inserted into the cuticular plate in control cultures after 2 days. C–G) After treatment with 20 μg/mL tumor necrosis factor (TNF)-α, general phalloidin-labeled stereocilia bundles appear disorderly by a immunofluorescence image (C), and collapse (D, E) and endocytosis (F, G) of the bundles into the hair cell body were observed. H) Mitochondria in control cultures contained normal outer, inner, and cristal membranes. The endoplasmic reticulum membrane was intact and smooth (arrow). I) Treatment of cultures for 2 d with 20 μg/mL tumor necrosis factor (TNF)-α resulted in enlargement, vacuolation and fission of mitochondria in outer hair cells. J) TNF-α treatment also caused swelling of the endoplasmic reticulum, and rough endoplasmic reticulum was degranulated (arrow). K) The tight junction (arrow) in the control cultures showed a normal electron density. L) Tight junction integrity was disrupted and with a hazy electron density (arrow) after 2 d with 20 μg/mL TNF-α. Scale bars: 0.20 μm.

TEM revealed orderly arranged bundles with the central filaments inserted straightly into the cuticular plate in the control group after 2 d in culture (Fig 2A and 2B). In comparison, the microarchitectural organization of the bundles was disturbed after treatment with 20 μg/mL TNF-α (Fig 2C), with collapse (Fig 2D and 2E) and endocytosis of stereocilia into the cell body (Fig 2F and 2G).

### TNF-α-induced ultrastructural changes

TEM of control OHCs cultured for 2 d revealed healthy mitochondria with normal outer, inner, and cristal membranes (Fig 2H). All these mitochondria were small and intact. In contrast, treatment with 20 μg/mL TNF-α for 2 d induced mitochondrial swelling, vacuolation, and fission (Fig 2I). The mitochondrial matrices appeared swollen, loose, and with reduced electron density, and cristae were broken, indicative of apoptosis.

Endoplasmic reticulum and their ribosomal components could be clearly seen by TEM in hair cells cultured for 2 d (Fig 2H, arrow). However, after exposure to 20 μg/mL TNF-α for 2 d, the endoplasmic reticulum appeared strikingly swollen, and the rough endoplasmic reticulum was degranulated (Fig 2J, arrow), indicative of endoplasmic reticulum stress responses.

In control cultures, the tight junction region formed between adjacent cell membranes was separated by intercellular space (Fig 2K, arrow). The tight junction was disrupted by 2 d treatment with 20 μg/mL TNF-α, where TEM showed a hazy structure with high electron density (Fig 2L, arrow).

### TNF-α-induced OHC extrusion and supporting cell contraction

**Fig 3:**
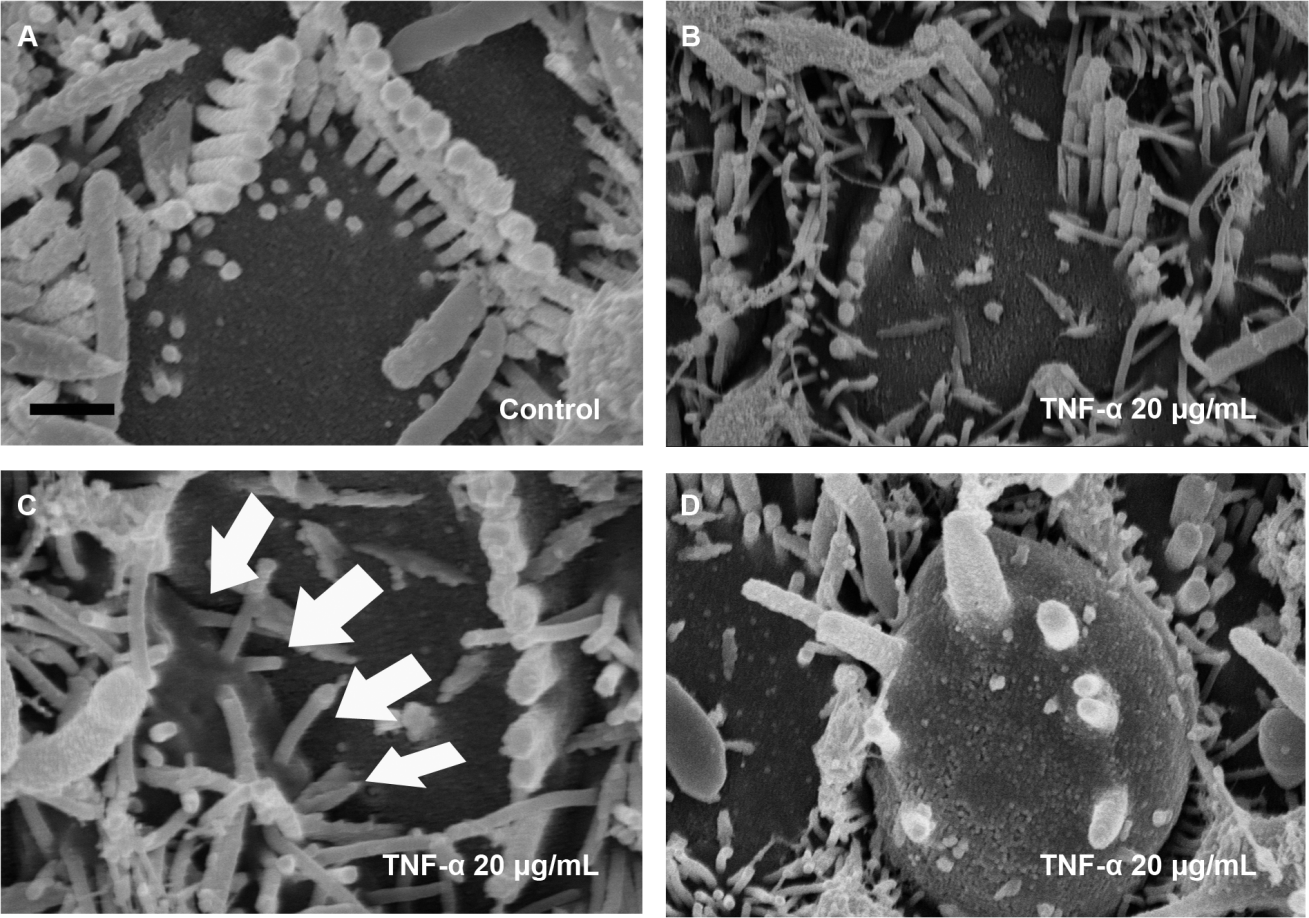
Scanning electron microscopy image of outer hair cell (OHC) extrusion and supporting cell contraction. A) Stereocilia bundles of control cultures remain orderly after 4 d. B–D) Treatment with tumor necrosis factor-α resulted in severely truncated OHC stereocilia bundles. C) The supporting cell was attempting to seal the pericuticular plate (arrow). D) OHCs appeared to undergo extrusion from the epithelium. Scale bar: 1 μm.

SEM also revealed that TNF-α treatment (20 μg/mL for 4 d) severely truncated OHC stereocilia bundles (Fig 3B and 3D). The bundle roots remained on the cuticular plate, while the cuticular plate of OHCs was swollen. The abundance of microvilli indicates that stereocilia bundles were more vulnerable than microvilli to TNF-α. The supporting cells were attempting to seal the pericuticular plate (Fig 3C, arrow) for restoration of the epithelial barrier. OHCs appeared to undergo extrusion from the epithelium (Fig 3D).

### TNF-α-induced apoptosis of supporting cells

**Fig 4:**
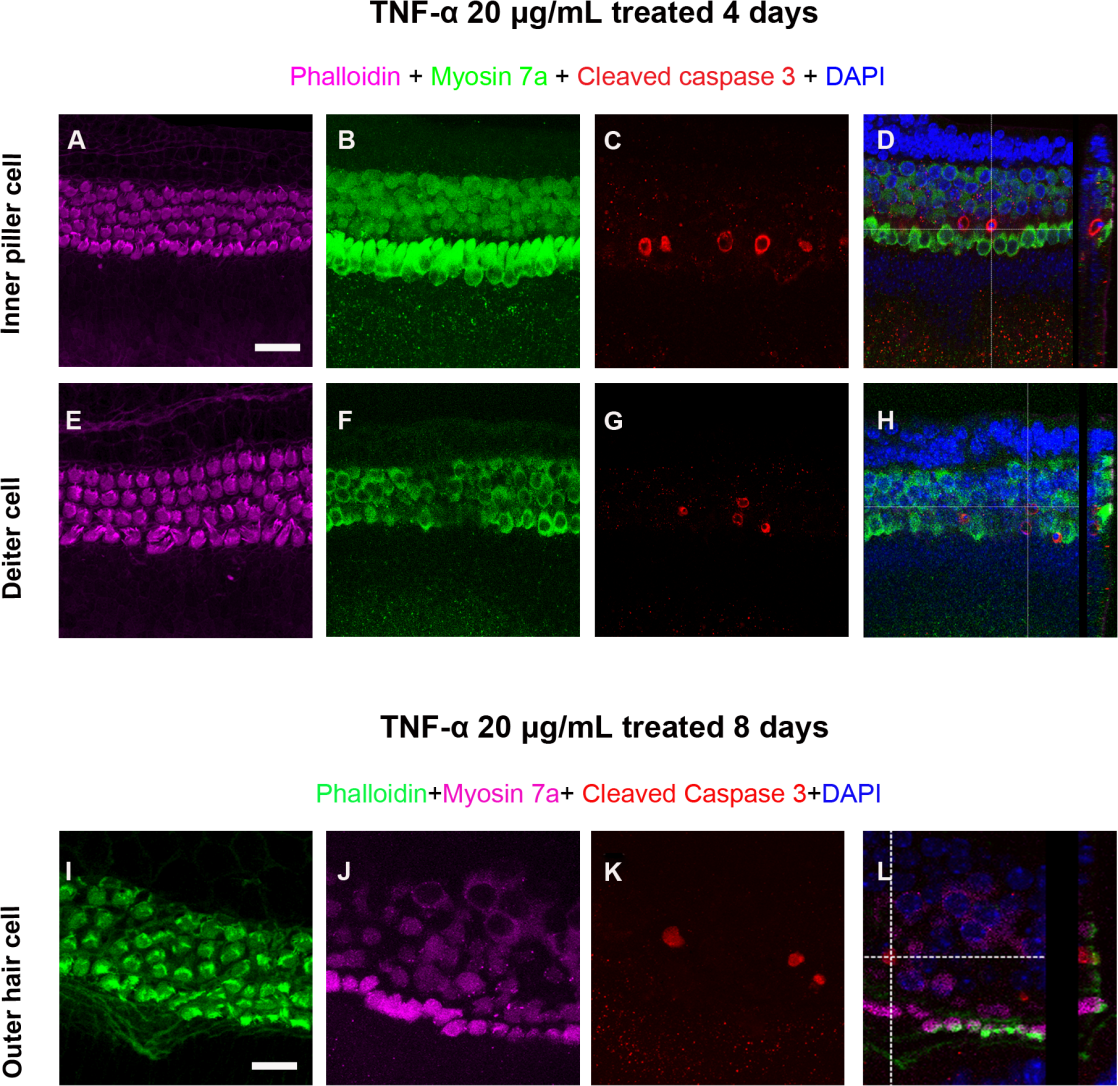
Cleaved caspase-3 in supporting cells and outer hair cells. A–H) Immunohistochemical staining showed that supporting cells, including inner pillar cells (C) and Deiters’ cells (G), were positive for apoptosis-associated caspase-3 after exposure 20 μg/mL tumor necrosis factor-α for 4 d. D,H) Side views of reconstructed confocal images provided a clear view of labeled supporting cell subtypes. I–L) Caspase-3-positive cells were detected in outer hair cells after exposure to 20 μg/mL tumor necrosis factor-α for 8 d. L) Side view of reconstructed confocal images. Scale bars: 50 μm.

Cleaved caspase-3 was detected by immunohistochemistry in cultures after treatment for 4 d with 20 μg/mL TNF-α (Fig 4A–4H). In particular, supporting cells (including inner pillar and Deiters’ cells), but not hair cells, were immunoreactive for cleaved caspase-3 (Fig 4D and 4H), indicating they underwent caspase signaling events associated with the induction of apoptosis.

### TNF-α-induced hair cell loss

**Fig 5:**
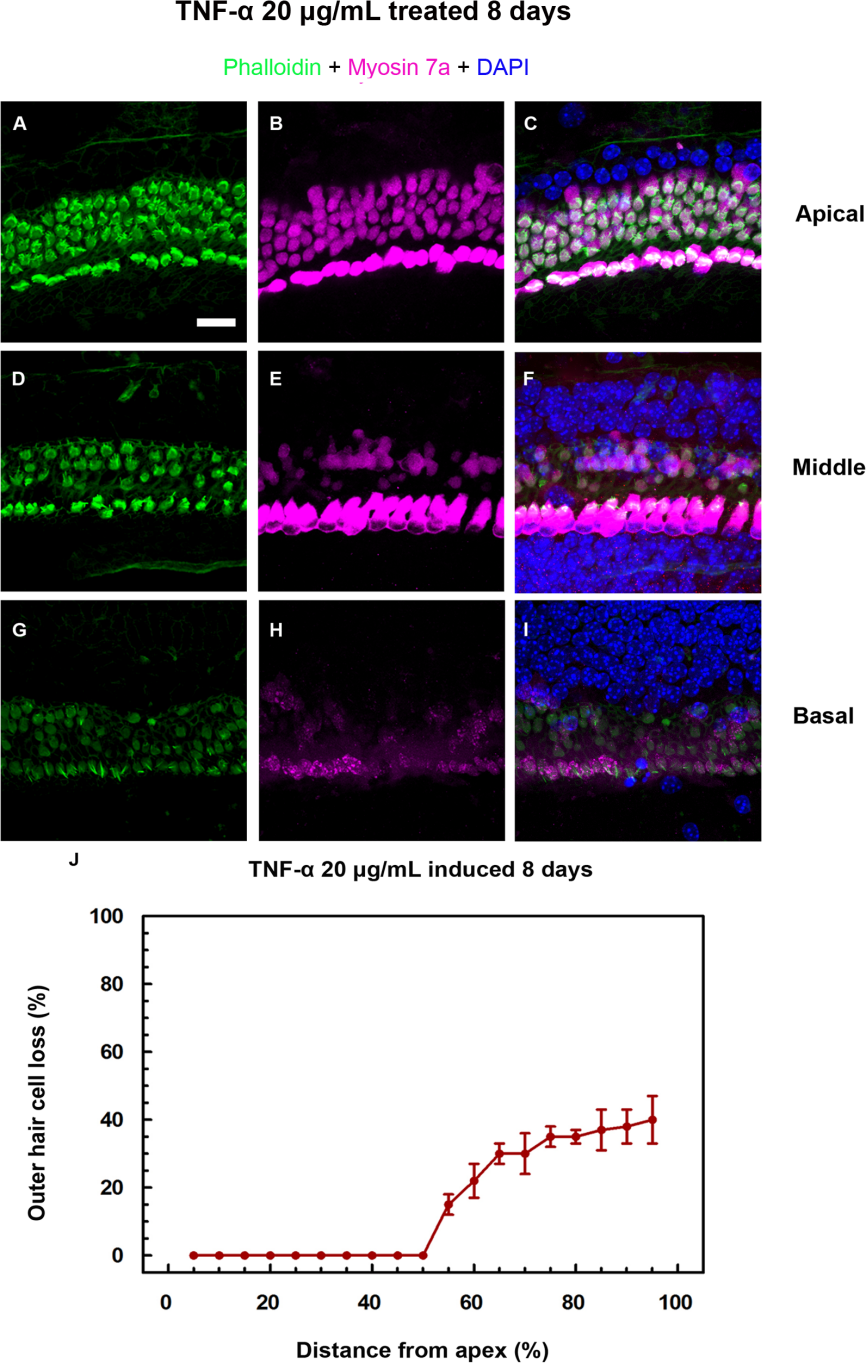
Immunofluorescence depicting outer hair cell death and Cochleogram of outer hair cell loss. A–C) Although the stereocilia bundles were disorganized at the apical turn of the cochlea, the hair cell bodies were intact. D–F) Loss of cell bodies and stereocilia at the cuticular plate was observed at the middle turn. G–I) The loss of hair cells and stereocilia was most pronounced at the basal turn. G) Some bundles and the cuticular plate disappeared, and stereocilia of inner hair cells fused into giant stereocilia. J) Cell loss after exposure to 20 μg/mL tumor necrosis factor-α for 8 d was greatest in the middle and basal turn region. Approximately 40% loss of outer hair cells was counted in the middle and basal turn region. Labeling: phalloidin, stereocilia bundles; myosin 7a, cell bodies; DAPI, nuclei. Scale bar: 50 μm.

Cleaved caspase-3 immunostaining was also detected in OHCs at the apical turn of the organ of Corti after 8 d of treatment with 20 μg/mL TNF-α (Fig 4I–4L). In the apical turn area of the cochlea, stereocilia bundles and cell bodies were intact (Fig 5A–5C), though the stereocilia bundles were disorganized (Fig 5A). At the middle turn of the cochlea, the loss of hair cell bodies and stereocilia was evident at the cuticular plate area (Fig 5D and 5E). The damage degree at the basal turn was more severe than the middle turn (Fig 5G–5I). OHC cell loss was quantified as a percentage on a cochleogram (Fig 5J). At the highest concentration (20 μg/mL TNF-α), treatment for 8 d resulted in approximately 40% loss of OHCs in the region 50–100% of the distance from the apex. Little or no OHC loss was seen in normal control groups cultured for 2 or 4 d; with < 5% loss in the region 70–100% of the distance from the apex after 8 d (data not shown).

## Discussion

This study describes the TNF-α-induced morphologic and ultrastructural changes in the organ of Corti. The findings demonstrate dose- and time-dependent damage, that was most pronounced in the basal turn area of the explants, consistent with the results by Haake et al. [12]. The increased vulnerability of the basal turn of the cochlea is also consistent with aminoglycoside- and cisplatin-induced ototoxicity [17].

### Disruption of hair cell stereocilia, mitochondrial and endoplasmic reticulum

Stereocilia disorganization was the initial sign of TNF-α-induced damage and fusion. Hair cell stereocilia are apical membrane protrusions comprised of uniformly polarized actin filament bundles. The actin paracrystal is tapered at the base of the bundles, and the majority of filaments terminate in close positions to the plasma membrane, leaving only the central filaments to insert in the cuticular plate. The stereocilia bundle is vulnerable to mechanical and ototoxic drug insults because of its delicate microarchitecture and membrane proteins, including ion channels, chemoreceptors, and cell adhesion molecules [18, 19]. The initial central disruption of the V formation may be an early sign of degeneration of the bundle before fusion and loss of stereocilia bundles. This is where the kinocilium is located. The kinocilium acts as a sensor for the establishment of the planer polarity within the cochlear epithelium and plays a key role in bundle development. So we speculate that the kinocilium and stereocilia in the center of the V formation may be active in actin protein turnover. While the organelles failed to synthesize proteins for the turnover activity due to noxious TNF-α exposure, the degeneration started from the location of the kinocilium. Our study used multiple visualization methods, including fluorescent-labeled phalloidin, TEM, and SEM, to show that increasing the dose and duration of TNF-α treatment induces stereocilia bundle collapse, fusion, truncation, and loss.

It is not entirely clear whether the initial damage is a direct result of TNF-α ototoxicity or secondary to the changes in mitochondria, such as enlargement, vacuolation and fission, and/or the endoplasmic reticulum, including edema and degranulation. Mitochondria are a major target of inflammation-associated injury, and TNF-α can induce suppression of complexes I and IV and pyruvate dehydrogenase activities [20] and cause mitochondrial damage [13]. Mitochondrial oxidative phosphorylation is the primary source of cellular energy, and is important for the transportation of actin and other molecules necessary for renewal of the stereocilia bundle. A previous study showed that the structure and length of the hair bundles are continuously maintained by actin filament treadmilling, with complete turnover every 48 hours [21, 22]. Thus, mitochondrial and endoplasmic reticulum abnormalities could disrupt the synthesis and transport of actin and other molecules, resulting in degeneration of the stereocilia bundles. Damage of the stereocilia bundle in mammalian hair cells can not be spontaneously repaired, although tip-link disruption can be self-repaired [23]. Nevertheless, damage to the stereocilia will affect mechanotransduction, which will lead to hearing loss.

### OHCs are more vulnerable to TNF-α treatment than IHCs

We show that TNF-α treatment resulted in loss of approximately 40% of OHCs in the basal turn. Although IHC damage (such as damage in the stereocilia bundles) was also observed, the damage to OHCs was more extensive and severe, consistent with other findings [24, 25]. Although the mechanism of this differential vulnerability is not clear, it is possible that the abundance of mitochondria in OHCs, which are one of main targets of TNF-α ototoxicity, is a contributing factor. In addition, examination of IHC and OHC transcriptomes by Liu et al. [26] showed that IHCs have stronger expression of several members of the *BCL* gene family, which are one of the determining factors for anti-apoptosis. Moreover, it is possible that OHCs may contain more TNF receptors than IHCs do. All these possible mechanisms may explain why IHCs are more resilient to insults than OHCs.

### Apoptosis of supporting cells precedes apoptosis of hair cells

Supporting cells serve important functions in the development, survival, death, phagocytosis, and regeneration of other cell types within the inner ear [27]. However, few studies have examined damage to supporting cells during inflammatory responses. Our SEM findings suggest that TNF-α treatment induces supporting cells to seal the reticular lamina. Thus, some supporting cells may exert their function to maintain the endolymph and perilymph separation in order to reduce hair cell damage. However, pillar and Deiters’ cells were also vulnerable to TNF-α, showing evidence of caspase-3 activation, which was not observed in OHCs until eight days. Thus, it appears that the activation of apoptosis-related signaling pathways in supporting cells preceded that of hair cells. Supporting cells are typically more resistant to other types of damage in comparison to hair cells, which makes this finding interesting. A possible explanation for this observation could involve the relative differential expression of TNF receptor 1 between supporting cells and hair cells. A previous study [1] shows that no TNF receptor 1 expression is detected in the normal cochlea by immunohistochemical staining. However, a weak TNF receptor 1 staining was found in supporting cells after intense vibration. A recent transcriptomic analyses of inner and outer hair cells respectively show that the *TNF receptor super family 1a* gene (*TNFRSF1a*) and the *TNFRSF1b* gene are expressed in both IHCs and OHCs, but the expression is very close to the baseline intensity level [26]. These two studies support the idea that both hair cells and supporting cells express TNF receptor 1, and that supporting cells may have more expression of TNF receptor 1, which would cause supporting cells to be more vulnerable to TNF-α.

### Degree of inflammatory cytokine-induced hair cell damage and loss

Confocal microscopy revealed a loss of stereocilia and the cuticular plate with TNF-α treatment. Hair cell loss can occur by sub-laminal and extrusion [28, 29], for which evidence was provided in our studies using SEM. These two modes of hair cells loss are consistent with the results of two other studies [30, 31].

In our experiments, treatment of cochlear explants with a high concentration of TNF-α (20 μg/mL) for eight days led to approximately 40% loss of OHCs. In contrast, Haake et al. [12] found that hair cells loss occurred with a much lower concentration and with a shorter duration of treatment (2 μg/ml TNF-α for four days). There are two possible explanations for this discrepancy. First, from the image they presented, it appears that the explant in their studies was only loosely attached to the bottom of the dish. This can affect the normal growth of hair cells, possibly leaving them more vulnerable to ototoxic drug treatment. Second, although TNF receptors are expressed in all tissues, reaction to stimulation can differ among various animal models. For example, Park et al. [13] showed that treatment with 30 ng/mL of TNF-α (with interleukins 1β and 6) for 48 hours decreased the viability of a mouse cochlear cell line. Although these studies had different specific time points and concentrations for measurements, they are roughly in agreement concerning the damage to cochlear tissues.

Compared with aminoglycoside-induced ototoxicity [32], treatment with TNF-α results in a moderate degree of damage to hair cells. This disparity may result from differences in the mechanisms of action. Endocytosis and ion channel transport both mediate the uptake of aminoglycosides into sensory hair cells, however, there is also strong evidence of entry through the mechanoelectrical transducer channels located at the top of the stereocilia [33]. In contrast, TNF-α binds to and induces receptor trimerization, which in turn triggers signaling cascades to exert hair cell death.

Previous work has indicated that TNF-α causes minimal hair cell death at concentrations used *in vivo* experiments [10]. We acknowledge that the concentration of TNF-α released by inflammatory responses *in vivo* is much lower than the concentrations used in our study. Moreover, culture conditions are different from the *in vivo* environment. However, we choose the *in vitro* model in order to more precisely control the concentrations and exposure duration to investigate TNF-α-induced ototoxicity. Therefore, extensive hair and supporting cell death was observed in our study. Nevertheless, with this model, we were able to investigate and detail the mechanism and pattern of proinflammatory cytokine-induced cochlear damage.

In summary, our study for the first time demonstrates the ultrastructural changes of organ of Corti after TNF-α treatment. TNF-α induces apoptosis of hair cells and supporting cells through caspase signaling. Treatment with high concentration and long duration of TNF-α results in a moderate degree of loss to hair cells. The present study provides an *in vitro* model of proinflammatory-induced ototoxicity.
